# Different Regulation of Glut1 Expression and Glucose Uptake during the Induction and Chronic Stages of TGFβ1-Induced EMT in Breast Cancer Cells

**DOI:** 10.3390/biom10121621

**Published:** 2020-12-01

**Authors:** Azadeh Nilchian, Nikolina Giotopoulou, Wenwen Sun, Jonas Fuxe

**Affiliations:** Karolinska Institutet, Department of Laboratory Medicine (LABMED), H5, Division of Pathology, F46, Karolinska University Hospital, 141 52 Huddinge, Sweden; azadeh.nilchian@ki.se (A.N.); nikolina.giotopoulou@ki.se (N.G.); wenwen.sun@ki.se (W.S.)

**Keywords:** breast cancer, Glut1, glucose uptake, TGF-β1, epithelial-mesenchymal transition, EMT, cell proliferation

## Abstract

Transforming growth factor beta 1 (TGF-β1) is associated with epithelial-mesenchymal transition (EMT), lymph metastasis, and poor prognosis in breast cancer. Paradoxically, TGF-β1 is also a potent inhibitor of cell proliferation. TGF-β1-induced EMT involves activation of several pathways including AKT, which also regulates glucose uptake. Recent data show that prolonged TGF-β1 exposure leads to a more stable EMT phenotype in breast cancer cells. However, whether this is linked to changes in glucose metabolism is not clear. Here, we used a model of TGF-β1-induced EMT in mammary epithelial cells to study the regulation of Glut1 and EMT markers during the induction compared to a prolonged phase of EMT by western blot, immunofluorescence and qPCR analysis. We also measured cell proliferation and uptake of the glucose analogue 2-NDBG. We found that EMT induction was associated with decreased Glut1 expression and glucose uptake. These effects were linked to reduced cell proliferation rather than EMT. Knockdown of Glut1 resulted in growth inhibition and less induction of vimentin during TGF-β1-induced EMT. Intriguingly, Glut1 levels, glucose uptake and cell proliferation were restored during prolonged EMT. The results link Glut1 repression to the anti-proliferative response of TGF-β1 and indicate that re-expression of Glut1 during chronic TGF-β1 exposure allows breast cancer cells to develop stable EMT and proliferate, in parallel.

## 1. Introduction

Malignant progression of breast cancer is associated with re-activation of epithelial-mesenchymal transition (EMT), a latent developmental process [1,2]. Breast cancer cells undergoing EMT lose epithelial characteristics including cell–cell junctions and apical-basal polarity. In parallel, cells acquire the expression of mesenchymal proteins including intermediate filaments like vimentin, which enhances their invasive and metastatic capabilities.

Induction of EMT can be triggered by inflammatory cytokines that are overexpressed in the tumor microenvironment. The cytokine transforming growth factor beta 1 (TGF-β1), which is a potent inducer of EMT, is upregulated in breast cancer tissues and associated with lymph metastasis and poor prognosis [3]. Thus, TGF-β1-induced EMT represents a link between cancer and inflammation [4]. On the other hand, TGF-β1 is also a potent inhibitor of cell proliferation and a maintenance factor for tissue homeostasis. TGF-β1 is therefore often described as having dual, paradoxical roles in cancer—on one hand, by acting as a tumor suppressor by inhibiting cell proliferation, and—on the other hand, by promoting EMT, cellular invasion and metastasis [3]. Various studies have been performed to elucidate the mechanisms of these somewhat conflicting roles of TGF-β1 in cancer [5]. 

TGF-β1-induced EMT involves transcriptional reprogramming, which is mediated by EMT-promoting transcriptional complexes formed by SMADS and core EMT transcription factors (EMT-TFs) including members of the SNAIL, ZEB and TWIST families [6,7]. Activation of such EMT-promoting complexes results in transcriptional inhibition of epithelial genes, and activation of genes promoting a mesenchymal phenotype. The induction of EMT in response to TGF-β1 is reversible in many cellular model systems. However, recent data show that prolonged exposure to TGF-β1 stabilizes the EMT program and allows tumor cells to develop more invasive and metastatic properties [8]. 

Importantly, mechanisms exist that act to preserve the epithelial phenotype and protect against EMT. For example, the enzyme glucose synthase kinase 3 beta (GSK-3β), a downstream target of AKT/PKB, which is active in epithelial cells, targets SNAIL and ZEB factors for degradation. Thus, even if these core EMT-TFs may be transcriptionally induced, they are degraded by GSK-3β. In contrast, hyperactivation of AKT-one of the most frequently observed signaling disturbances in tumor cells, leads to inactivation of GSK-3β, which allows SNAIL and ZEB to be active and promote EMT. Recently, we identified the coxsackievirus and adenovirus receptor (CXADR), a tight junction-based transmembrane protein, acts as a negative regulator of the AKT signaling pathway by forming a protein complex with the AKT inhibitors PTEN and PHLPP2. Loss of CXADR, which is detected during malignant progression of breast cancer and other tumors of epithelial origin, leads to hyper-activation of AKT, inhibition of GSK-3β, increased activation of SNAIL and TWIST and more pronounced EMT in response to TGF-β1 [9]. 

The AKT pathway is also linked to EMT through its central role in regulating glucose metabolism. High levels of glucose have been shown to promote EMT in breast cancer cells [10,11]. Moreover, inhibition of GSK-3β results in upregulation of the glucose transporter Glut1 and increased cellular uptake of glucose [12]. Yet, the role of Glut1 in regulating TGF-β1-induced EMT is less clear. In particular, it is not known to what extent Glut1 expression and glucose uptake plays a role in the acquisition of a more stable EMT phenotype. To elucidate this, we studied Glut1 regulation and glucose uptake in an established model of TGF-β1-induced EMT in mammary epithelial cells. Based on previous studies showing that EMT is induced during the first 72 h, and that cells enter into a more stable EMT phenotype after 14 days, we selected these time points for our studies [8,13]. The results showed that Glut1 levels and glucose uptake are differently regulated during the induction phase compared to a more stable TGF-β1-induced EMT. 

## 2. Materials and Methods

### 2.1. Cell Lines and Cell Culture Conditions

Namru Murine Mammary gland (NMuMG) cells (ATCC, Teddington, UK) were cultivated in DMEM with high glucose (4500 mg/L) (Thermo Fisher Scientific, Gothenburg, Sweden) supplemented with 10% FBS and 1% penicillin–streptomycin. EpH4 cells (originally provided by the lab of H. Beug, Vienna Medical University) were cultivated in DMEM/F-12 medium (Thermo Fisher Scientific) supplemented with 10% Fetal Bovine Serum (Thermo Fisher Scientific) and 1% penicillin–streptomycin. MDA-MB-231 and MCF7 cells (ATCC) were cultured in DMEM (Thermo Fisher Scientific) supplemented with 10% FBS and 1% penicillin–streptomycin. EMT was induced by the addition of recombinant mouse TGF-β1 (R&D Systems, Abingdon, UK) at a dose of 10 ng/mL for 72 h or 14 days. All cells were cultured at 37 °C in a 5% humidified CO_2_ incubator.

### 2.2. Western Blot Analysis

After treatment with or without TGF-β1, cells were lysed in RIPA buffer (#89900, Thermo Fisher Scientific) supplemented with protease and phosphatase inhibitors (#87785, Thermo Fisher Scientific). Total protein extracts were boiled in Bolt LDS sample buffer (#B0007, Thermo Fisher Scientific), separated by SDS–PAGE under reducing conditions and transferred to a nitrocellulose membrane using the Trans-Blot Turbo Transfer System (Bio-Rad, Solna, Sweden). Upon blocking in blocking reagent (#11520709001, Roche, Solna, Sweden) or 3% non-fat dry milk (Santa Cruz Biotechnology, CA, USA) for 1 h at room temperature, membranes were incubated with primary antibodies over night at 4 °C. The primary antibodies used were: rabbit anti-Glut1 (Merck/Millipore, Solna, Sweden); rabbit anti-AKT (Cell Signaling, Leiden, The Netherlands); rabbit anti-pAKT (cell signaling); rabbit anti-E-cadherin (Cell signaling), rabbit anti-Vimentin (Cell Signaling), each one diluted 1:1000. Membranes were then washed in PBS and incubated with appropriate rabbit or mouse HRP IgG (cell signaling) antibodies (diluted 1:2000) for 1 h at room temperature. All antibodies were diluted in blocking reagent (Roche). Immunoreactive bands were visualized by chemiluminescence (#WBLUF0100, Millipore, Solna, Sweden) and developed using an ChemiDoc XRS+ system with Image Lab Software (Bio-Rad), or an Odyssey Fc Imaging System (LI-COR Biosciences, Bad Homburg, Germany). 

### 2.3. Immunofluorescence Staining

Cells were grown on cover slips and fixed shortly in ice-cold absolute acetone for 2 min. The cover slips were incubated for 1 h in blocking solution (5% donkey serum, and 0.2% BSA in PBS). Primary antibodies were added and incubated at 4 °C overnight. Primary antibodies used were: rabbit anti-Glut1 (Merck/Millipore) and mouse anti-E-cadherin (BD Biosciences, San José, CA, USA) that were both diluted 1:1000. Cells were then washed in PBS containing 0.2% BSA and incubated in corresponding secondary antibodies for 1 h at room temperature. Secondary antibodies used were: anti-rabbit IgG (H + L) Alexa Fluor 594 and anti-mouse IgG (H + L) Alexa Fluor 488 (Thermo Fisher Scientific) that were diluted 1:700. The cover slips were mounted on Vectashield containing DAPI (Vector Labs, Burlingame, CA, USA). Images were captured by a Nikon Eclipse E800 microscope (Bergman Labora, Danderyd, Sweden) and Zeiss LSM700 confocal microscope (Zeiss, Stockholm, Sweden).

### 2.4. Glucose Uptake Measurement

To quantify glucose uptake the commercially available fluorescent glucose probe 2-NBDG (2-(*N*-(7-Nitrobenz-2-oxa-1,3-diazol-4-yl)Amino)-2-Deoxyglucose (#N13195, Thermo Fisher Scientific) was used according to the manufacture instruction. Briefly, cells were washed with warm (37 °C) PBS before incubation with the compound for 20 min at 37 °C. The cells were then washed with cold (4 °C) PBS to decrease metabolic activity before taking pictures with a Nikon Eclipse TE300 inverted microscope (Bergman Labora). For each condition cell were sided in 4 replicas and a minimum of 4 images were taken per well. All experiments were carried out for at least 3 independent times. Images were analyzed using Image J software (https://imagej.nih.gov/ij/). 

### 2.5. Quantitative Real-Time PCR Analysis

Total RNA was extracted using the RNeasy mini kit (Qiagen, Valencia, CA, USA) according to the manufacturer’s instructions. cDNA synthesis was performed by using the QuantiTect Reverse Transcription kit (Qiagen) using 1 μg of total RNA. For qPCR (quantitative realtime PCR) analysis, 5 ng of the cDNA mixture was used for PCR amplification by QuantiFast SYBR Green PCR Kit (Qiagen) with validated QuantiTect primers (Qiagen). The following genes were analyzed: *Glut1*, *CXADR*, *Vimentin* and *L19*. The PCR was carried out as follows: 3 min at 95 °C followed by 35 cycles of 3 s at 95 °C, 20 s at 55 °C and a 2 s extension step at 72 °C in ABI7500 (Thermo Fisher Scientific) PCR system. 

### 2.6. Proliferation Assays

60,000 cells were seeded in each of 4 wells in a 48 plate. Untreated or pretreated cells with TGF-β1 (10 ng/mL) (R&D Systems, Abingdon, UK) for 72 h were removed with Trypsin (Thermo Fisher Scientific) and stained with trypan blue solution (Thermo Fisher Scientific). The cells were counted, 3 times per well, in the Countess II Automated Cell Counter (Thermo Fisher Scientific). The same procedure was used for NMuMG, EpH4, MCF7 and MDA-MB-231 cells.

### 2.7. RNA Interference

200,000–500,000 NMuMG cells were seeded per well in a 6-well plate. The next day, cells were transfected with On-Targetplus siRNA Smartpool against Glut1/scrambled siRNA (Dharmacon/Thermo Fisher Scientific) using Lipofectamine RNAiMAX (Thermo Fisher Scientific) according to the manufacturer’s instructions in a final siRNA concentration of 10 µM. After 24 h, cells were trypsinized and 200,000 cells per well were seeded in a new 6-well plate. After another 24 h, cells were treated with TGF-β1 (10 ng/mL) (R&D Systems) for 72 h and whole cell lysates were harvested using RIPA buffer (Thermo Fisher Scientific).

### 2.8. Stable Overexpression of Glut1

100,000 NMuMG cells were seeded per well in a 6-well plate (Thermo Fisher Scientific). On the next day, cells were transduced with 20 μL lentiviral particles (Amsbio, Abingdon, UK) at 2 MOI (multiplicitiy of infection) units and incubated for 72 h. 5 µg/mL blasticidin (Thermo Fisher Scientific) was used for selection.

### 2.9. Statistical Analysis

Statistical analysis was performed using the Student’s t-test for comparisons between two groups or ANOVA followed by the Bonferroni post hoc test for multiple group analysis. Significant differences are indicated by: * (*p* < 0.05), ** (*p* < 0.01), *** (*p* < 0.001). 

## 3. Results

### 3.1. Reduced Glut1 Expression and Glucose Uptake during the Induction of EMT

**Figure 1:**
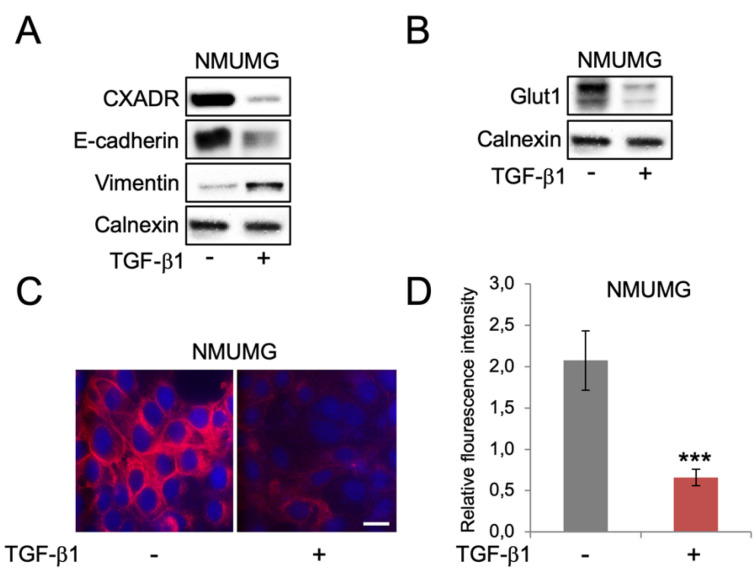
TGF-β1-induced EMT in NMuMG mammary epithelial cells is associated with repression of Glut1 and reduced glucose uptake. (**A**,**B**) Western blot analysis of the effects of short-term TGF-β1 exposure (10 ng/mL, 72 h) on the expression of the EMT markers CXADR, E-cadherin and vimentin (**A**), and Glut1 (**B**) in NMuMG cells. Calnexin was used as a loading control. (**C**) Immunofluorescence staining of Glut1 (red) in NMuMG cells that were non-treated or treated with TGF-β1 (10 ng/mL, 72 h). DAPI staining was used to visualize cell nuclei. Scale bar = 10 μm. (**D**) Bar graph showing the effects of TGF-β1 (10 ng/mL, 72 h) on uptake of 2-NBDG in NMuMG cells. *** *p* < 0.001.

A model of TGF-β1-induced EMT in Namru mouse mammary gland (NMuMG) epithelial cells was used to study Glut1 regulation and glucose uptake during the induction of EMT. Cells were maintained in high glucose (4500 mg/L) medium throughout the experiments. The epithelial markers E-cadherin and CXADR were repressed, while the mesenchymal marker vimentin was induced during the induction of EMT by TGF-β1, as expected (Figure 1A). Loss of CXADR and gain of vimentin were also detected by immunofluorescence staining (Supplementary Figure S1A), and at the mRNA level (Supplementary Figure S1B,C). Studies of Glut1 levels showed that Glut1 expression decreased during the induction of EMT, both at protein (Figure 1B,C) and mRNA levels (Supplementary Figure S1D). To study if this was associated with changes in glucose uptake we incubated cells with the fluorescent glucose analogue 2-NBDG. The results showed that uptake of 2-NBDG was inhibited during the induction of EMT (Figure 1D).

### 3.2. Reduced Expression of Glut1 and Glucose Uptake Is Linked to Decreased Cell Proliferation but not EMT

**Figure 2:**
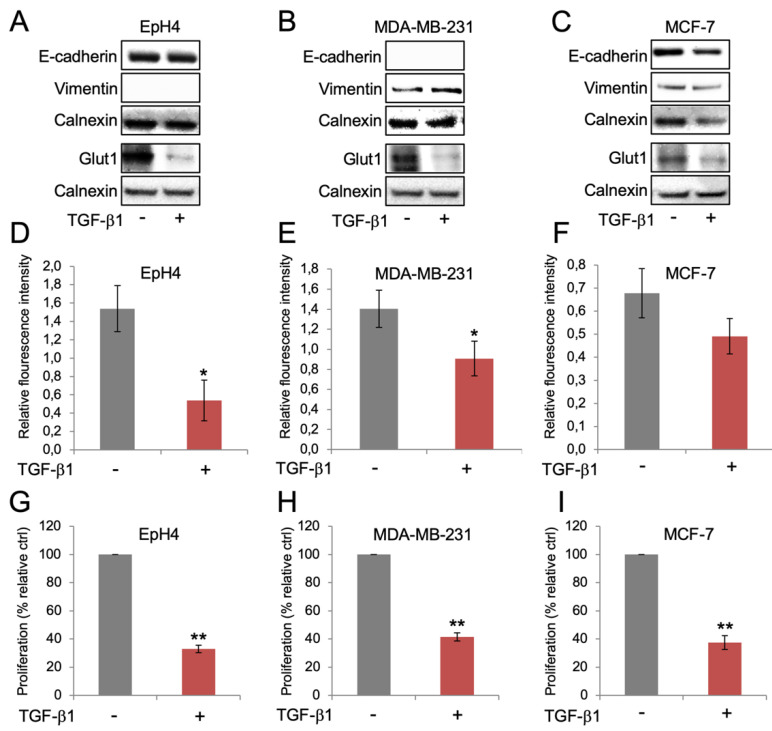
Repression of Glut1 in breast cancer cells by TGF-β1 is not specifically linked to an EMT response, but to reduced glucose uptake and growth inhibition. (**A**–**C**) Western blot analysis of the effects of TGF-β1 exposure (10 ng/mL, 72 h) on the expression of the EMT markers E-cadherin and vimentin, and Glut1 in EpH4 cells (**A**), MDA-MB-231 cells (**B**) and MCF-7 cells (**C**). Calnexin was used as a loading control. (**D**–**F**) Bar graphs showing the effects of TGF-β1 (10 ng/mL, 72 h) on the uptake of 2-NBDG in EpH4 cells (**D**), MDA-MB-231 cells (**E**) and MCF-7 cells (**F**). (**G**–**I**) Bar graphs showing the effects of TGF-β1 (10 ng/mL, 72 h) on the proliferation of EpH4 cells (**G**), MDA-MB-231 cells (**H**) and MCF-7 cells (**I**). * *p* < 0.05; ** *p* < 0.01.

The results prompted us to study whether the reduction of Glut1 expression and glucose uptake, which was observed during TGF-β1-induced EMT in NMuMG cells, was specifically linked to an EMT response. We therefore repeated the experiments in EpH4 cells, which are refractory to TGF-β1-induced EMT [14,15]. Treatment of EpH4 cells with TGF-β1 did not result in repression of E-cadherin or induction of vimentin (undetectable) (Figure 2A). Yet, Glut1 levels were reduced also in EpH4 cells after TGF-β1 treatment, suggesting that this effect was independent of an EMT response. To investigate this further we included MDA-MB-231 and MCF-7 cells, two human breast cancer cell lines that display mesenchymal and epithelial properties, respectively. TGF-β1 treatment resulted in repression of Glut1 in both MDA-MB-231 and MCF-7 cells, despite clear changes in E-cadherin or vimentin expression (Figure 2B,C). In agreement with this, we found that uptake of 2-NBDG was inhibited in EpH4 cells, MDA-MB-231 cells and MCF-7 cells after TGF-β1 treatment (Figure 2D–F). Based on the documented role of TGF-β1 in acting both as a tumor promoter by inducing EMT, and a tumor suppressor by inhibiting cell proliferation, we studied to what extent Glut1 repression was associated with growth inhibition in the different cells. The results showed that Glut1 repression was associated with reduced cell proliferation in EpH4 cells, MDA-MB-231 cells and MCF-7 cells after TGF-β1 treatment (Figure 2G–I). 

### 3.3. Re-Expression of Glut1 and Increased Glucose Uptake during Long-Term EMT

**Figure 3:**
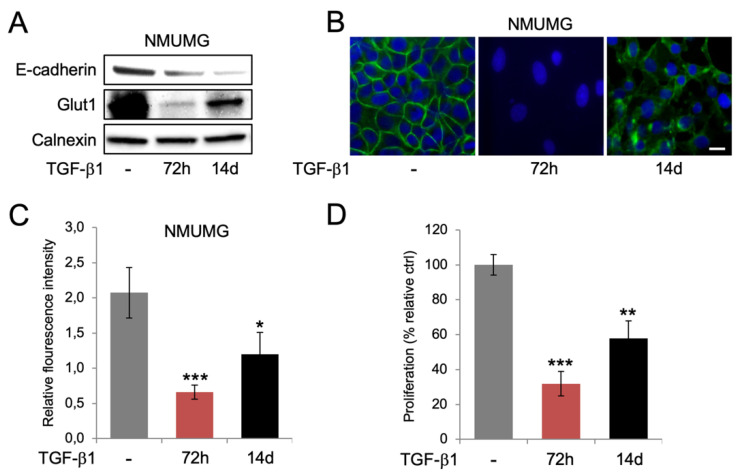
Prolonged TGF-β1 exposure is associated with more pronounced EMT, re-expression of Glut1, and increased glucose uptake and cell proliferation. (**A**) Western blot analysis of the expression of E-cadherin and Glut1 in NMuMG cells after short-term (72 h) and long-term (14 days) exposure to TGF-β1. Calnexin was used as a loading control. (**B**) Immunofluorescence staining of Glut1 in NMuMG cells after short-term (72 h) and long-term (14 days) TGF-β1 exposure. DAPI staining was used to visualize cell nuclei. Scale bar = 10 μm. (**C**,**D**) Bar graphs showing the effects of short-term (72 h) and long-term (14 days) exposure of NMuMG cells to TGF-β1 on the uptake of 2-NBDG (**C**) and cell proliferation (**D**). * *p* < 0.05; ** *p* < 0.01; *** *p* < 0.001.

Considering recent reports showing that prolonged TGF-β1 exposure promotes a more stable EMT phenotype and a more aggressive behavior of breast cancer cells, we were curious to study if there was a difference in the regulation of Glut1 expression and glucose uptake in cells during the induction of EMT compared to when they acquired a more stable TGF-β1 phenotype. To study this, we exposed NMuMG cells to TGF-β1 for either 72 h, or 14 days. Western blot analysis showed that E-cadherin levels were lower at 14 days compared to 72 h, indicating that the EMT effect by TGF-β1 was more pronounced at the longer time point (Figure 3A). In contrast, Glut1 levels were increased at 14 days compared to 72 h, which was also observed by immunofluorescence staining (Figure 3A,B). In line with this, we found that glucose uptake and cell proliferation was increased at 14 days compared to 72 h of TGF-β1 exposure (Figure 3C,D). No differences in p-AKT levels were detected between the 72 h and 14 days time points (Supplementary Figure S1E). 

### 3.4. Glut1 Regulates Cell Proliferation and TGF-β1-Induced EMT

**Figure 4:**
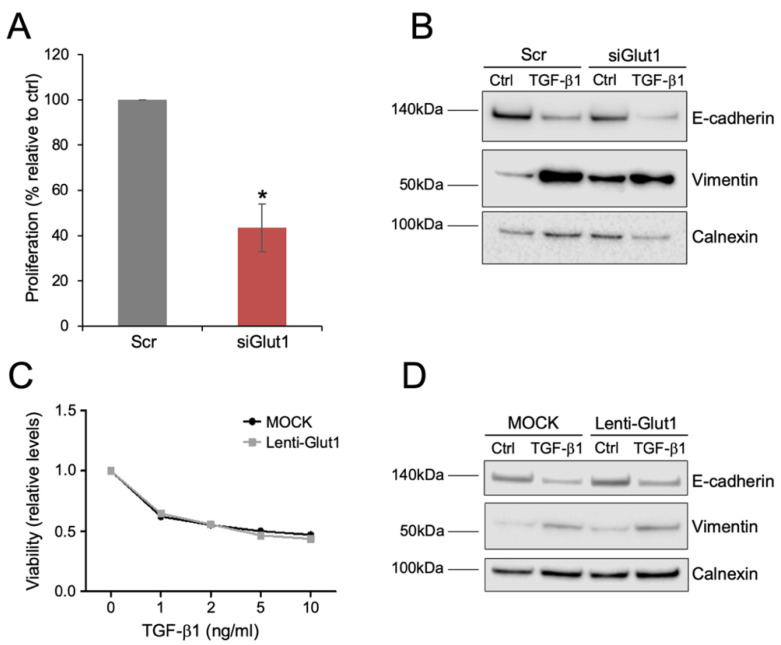
Glut1 regulates cell proliferation and TGF-β1-induced EMT in mammary epithelial cells. (**A**) Bar graph showing the effect of transection of scrambled control (Scr) or siRNA against Glut1 (siGlut1) on the proliferative capacity of NMuMG cells. (**B**) Western blot analysis showing the effect of scrambled control (Scr) or Glut1 siRNA (siGlut1) on TGF-β1-induced changes in the expression of E-cadherin and vimentin during the induction of EMT in NMuMG cells. (**C**) Line graph showing the effect of lentivirus-mediated overexpression of Glut (RFP-Glut1) or control (RFP) on the viability of NMuMG cells after exposure to different doses of TGF-β1 for 72 h. (**D**) Western blot analysis showing the effect of overexpression of Glut (RFP-Glut1) or control (RFP) on TGF-β1-induced changes in the expression of E-cadherin and vimentin during the induction of EMT in NMuMG cells. * *p* < 0.05.

The results indicated that Glut1 levels, although repressed during the induction of EMT, could play a role in regulating both proliferative and EMT responses. To study this, we performed loss- and gain-function experiments in NMuMG cells. Transfection of cells with *Glut1* siRNA resulted in reduced Glut1 protein levels compared to cells transfected with a scrambled control (Scr), as detected by immunofluorescence and western blot analyses (Supplementary Figure S2A,B). Knockdown of *Glut1* had a significant impact on cell proliferation, which was reduced compared to control cells (Figure 4A). We then studied whether knockdown of *Glut1* affected the EMT response downstream of TGF-β1. The morphological changes associated with the induction of EMT were clearly visible after TGF-β1 treatment and no obvious differences were observed between control cells and *Glut1* knockdown cells (Supplementary Figure S2C). Knockdown of *Glut1* had minor effects on the reduction of E-cadherin, but vimentin levels were less induced in *Glut1* knockdown cells compared to control cells after exposure to TGF-β1 (western blots in Figure 4B and statistical analysis in Supplementary Figure S2D,E).

Finally, we attempted to overexpress Glut1 in NMuMG cells via a lentivirus vector to study if cell proliferation and/or the induction of EMT was affected. Immunofluorescence staining showed more intense staining of Glut1 in areas of lenti-Glut1 transduced cells but western blot analysis did not indicate increased levels of Glut1 in lenti-Glut1 compared to mock transduced cells (Supplementary Figure S3A,B). Furthermore, lenti-Glut1 did not have a significant impact on the morphological changes or dose-dependent reduction of cell proliferation by TGF-β1 (Supplementary Figure S3B and Figure 4C); neither did lenti-Glut1 affect changes in E-cadherin or vimentin levels during TGF-β1-induced EMT (western blots in Figure 4D and statistical analysis in Supplementary Figure S3D,E).

## 4. Discussion

Changes in glucose metabolism is a hallmark of cancer progression and has been linked to TGF-β1-induced EMT. Recent data show that long-term TGF-β1 exposure leads to a more stable EMT phenotype in breast cancer cells. Here, we sought to investigate whether glucose uptake changed during different stages of TGF-β1-induced EMT in mammary epithelial cells. We found that during the induction phase of EMT, Glut1 was downregulated and accordingly, glucose uptake inhibited. However, loss of Glut1 and reduced glucose uptake was not specifically associated with an EMT response but was also detected in mammary tumor cells and breast cancer cells that were non-responders to TGF-β1-induced EMT. Instead, repression of Glut1 and glucose uptake was associated with the anti-proliferative effect of TGF-β1. Intriguingly, Glut1 levels, glucose uptake and cell proliferation increased as cells developed a more stable EMT phenotype during long-term exposure to TGF-β1. 

The results from short-term exposure to TGF-β1, which showed Glut1 repression and reduced glucose uptake, were somewhat surprising considering published reports showing that TGF-β1 promotes Glut1 expression and glucose uptake [16,17]. However, some of the discrepancies between those results and ours might be explained by the fact that fibroblasts, thus cells of a mesenchymal origin, were used as a model system in some of the earlier studies. Intriguingly, and in contrast to its effect on epithelial cells, TGF-β1 is known to stimulate proliferation of mesenchymal stem cells and fibroblasts, and to play an important role in fibrosis [18,19]. Together, our data and results from earlier studies indicate that the capacity of TGF-β1 to inhibit proliferation of epithelial cells, and stimulate proliferation of mesenchymal cells, is closely linked to its ability to either repress, or induce Glut1 expression, respectively. It will be interesting for future studies to elucidate to what extent Glut1 repression contributes to the anti-proliferative effect of TGF-β1 and how it is linked to previously known molecular mechanisms of TGF-β1-mediated growth inhibition including induction of the cell cycle inhibitors p15Ink4b, p21Cip1 and p27Kip1 [20]. It would also be interesting to explore the possible role of glucose and Glut1 in microcalcifications in the breast, which recently was linked to EMT and signaling by BMP2, another member of the TGF-β family [21,22]. 

Based on the different effects of TGF-β1 on the proliferation of epithelial vs. mesenchymal cells it was interesting to note that Glut1 expression bounced back as NMuMG cells acquired a more stable EMT phenotype. This was also associated with increased glucose uptake and enhanced cell proliferation. A possible interpretation of these results is that as epithelial cells develop a more accentuated mesenchymal phenotype during the time course of EMT, they gradually lose the capacity to become growth inhibited, and instead develop the capacity to become stimulated by TGF-β1. Thus, prolonged TGF-β1 exposure not only promotes more pronounced EMT but also activates a metabolic switch that allows breast cancer cells to escape the growth inhibitory effect of TGF-β1, and thereby co-develop invasive and proliferative capabilities. It will be interesting to further study to what extent Glut1 and possibly also other glucose transporters contribute to the metabolic switch during TGF-β1-induced EMT.

It will also be of importance to elucidate which signaling pathways play a major role in mediating the switch. We did not find any difference in p-AKT levels between the 72 h and the 14 days time points, suggesting that other pathways are involved. Others have found that EGFR/MAPK and integrin β1/Src/FAK pathways are regulated by Glut1 [23], suggesting that these pathways could play a role in the metabolic switch during TGF-β1-induced EMT. Further studies using a combination of metabolomics and in vivo experiments are needed to elucidate the signaling and molecular components of the metabolic switch, and how it affects the capacity of breast cancer cells to metastasize.

## 5. Conclusions

The results link Glut1 repression and reduced glucose uptake to the anti-proliferative response of TGF-β1 and indicate that re-expression of Glut1 and increased glucose uptake during chronic TGF-β1 exposure allow breast cancer cells to develop a more stable EMT and proliferate, in parallel. This provides evidence that prolonged TGF-β1-induced EMT in breast cancer cells is associated with a metabolic switch, which may be used as a diagnostic tool and/or therapeutic target for further translational studies.

## Supplementary Materials

The following are available online at https://www.mdpi.com/2218-273X/10/12/1621/s1, Figure S1: Regulation of EMT markers and Glut1 during TGF-β1-induced EMT in mammary epithelial cells, Figure S2: Effect of Glut1 knockdown on EMT, Figure S3: Effect of Glut1 overexpression on EMT, Figures S4: Original western blots.
