# Supplementary material for: Different Regulation of Glut1 Expression and Glucose Uptake during the Induction and Chronic Stages of TGFβ1-Induced EMT in Breast Cancer Cells
Source: Biomolecules. 2020 Dec 1;10(12):1621. doi: 10.3390/biom10121621 (PMC7760794; doi:10.3390/biom10121621)
Supplement: Supplementary file 1 [file biomolecules-10-01621-s001.pdf]

Supplementary Materials

Article

Different Regulation of Glut1 Expression and Glucose Uptake during the Induction and Chronic Stages of TGFβ1-Induced EMT in Breast Cancer Cells

Azadeh Nilchian †, Nikolina Giotopoulou †, Wenwen Sun and Jonas Fuxe *

Karolinska Institutet, Department of Laboratory Medicine (LABMED), H5, Division of Pathology, F46, Karolinska University Hospital, 141 52 Huddinge, Sweden; azadeh.nilchian@ki.se (A.N.); nikolina.giotopoulou@ki.se (N.G.); wenwen.sun@ki.se (W.S.)

* Correspondence: jonas.fuxe@ki.se; Tel.: +46707980065

† These authors contributed equally to this paper.

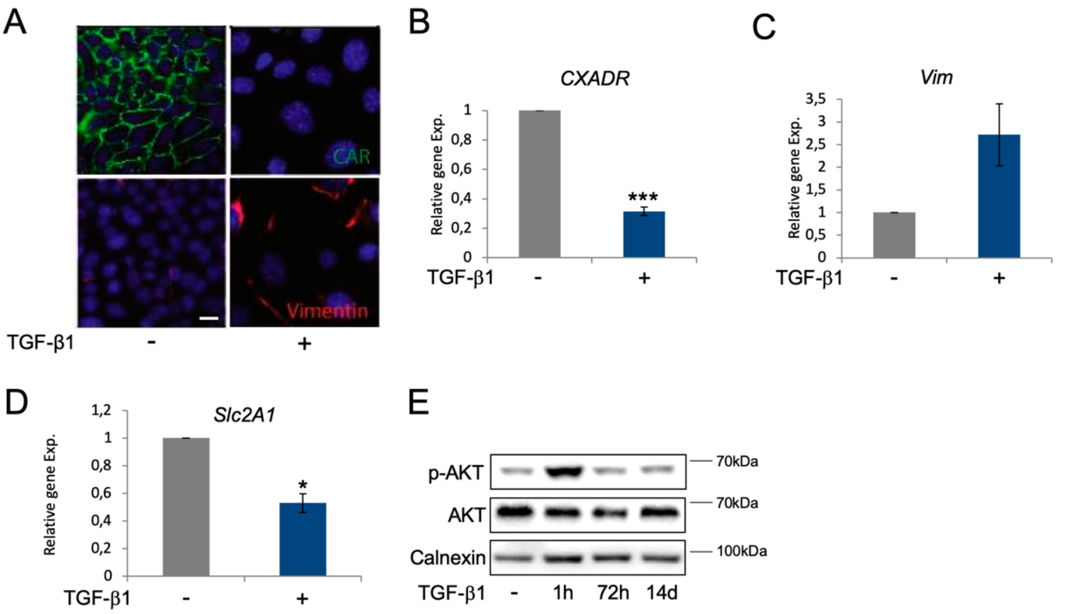

Figure S1. Regulation of EMT markers and Glut1 during TGF-β1-induced EMT in mammary epithelial cells. (**A**) Immunofluorescence staining of CXADR (green) and vimentin (red) in NMuMG cells after 72 h of TGF-β1 exposure (10ng/ml). DAPI staining was used to visualize cell nuclei. Scale bar = 10µm. (**B-D**) Bar graphs showing qPCR results from analyzing the effect of TGF-β1 exposure (10ng/ml) for 72 h on mRNA expression of *Cxadr*, *Vim* and *Slc2a1* (Glut1) in NMuMG cells. Data represent means±s.e.m. with three independent experiments in triplicates. **p*<0.05; ****p*<0.01.

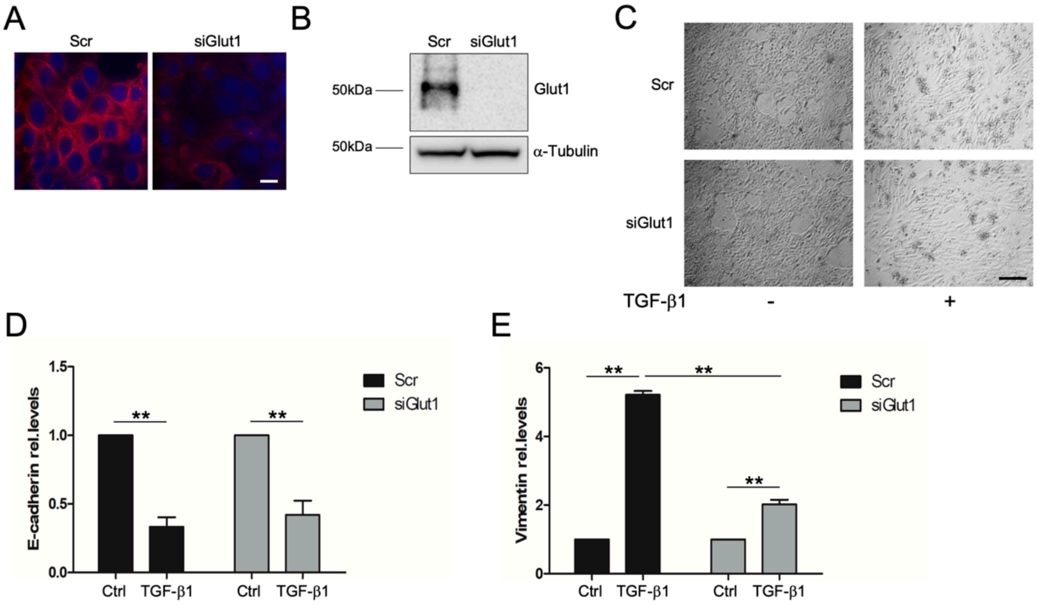

Figure S2. Effect of Glut1 knockdown on EMT. (**A**) Immunofluorescence staining of Glut1 (red) in NMuMG cells after transfection of scrambled control (Scr) or Glut1 siRNA (siGlut1). DAPI staining was used to visualize cell nuclei. Scale bar = 10µm. (**B**) Western blot analysis of the effect of scrambled control (Scr) or Glut1 siRNA (siGlut1) on Glut1 levels. α-Tubulin was used as a loading control. (**C**) Bright field images showing the effect of scrambled control (Scr) or Glut1 siRNA (siGlut1) on morphological changes associated with TGF-β1-induced EMT. Scale bar = 100µm. (**D, E**) Bar graphs showing quantification of western blot analysis of the effect of scrambled control (Scr) or Glut1 siRNA (siGlut1) on TGF-β1-mediated changes in E-cadherin (**D**) and vimentin (**E**) levels. Data represent means±s.e.m. with three independent experiments in triplicates. **p<0.01.

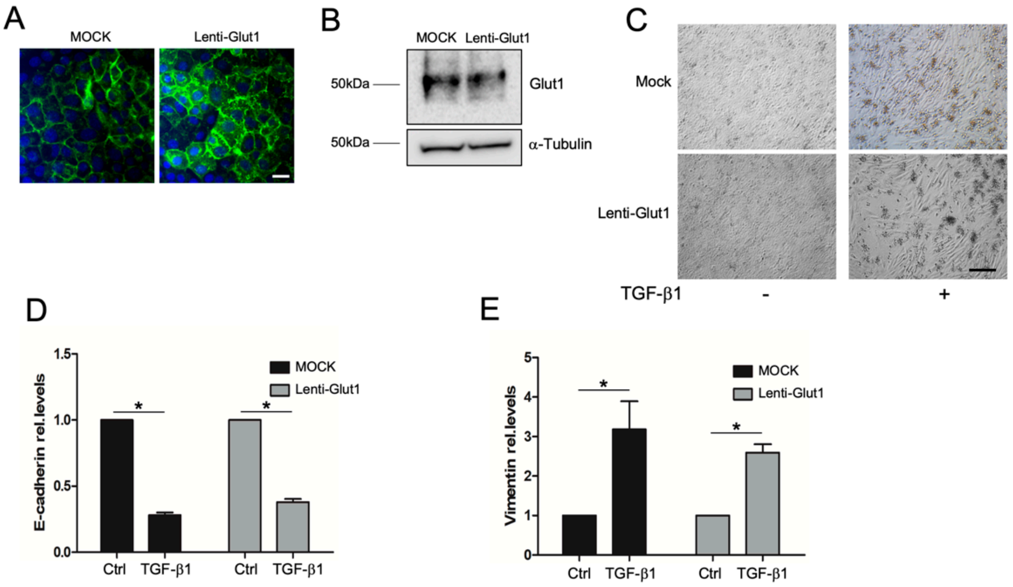

Figure S3. Effect of Glut1 overexpression on EMT. (**A**) Immunofluorescence staining of Glut1 (green) in NMuMG cells transduced with a lentivirus expressing non-coding cDNA (mock) or Glut1 (Lenti-Glut1). DAPI staining was used to visualize cell nuclei. Scale bar = 10µm. (**B**) Western blot analysis of Glut1 levels in NMuMG cells transduced with mock or Lenti-Glut1. α-Tubulin was used as a loading control. (**C**) Bright field images showing the effect of mock or Lenti-Glut1 transduction on morphological changes during TGF-β1-induced EMT. Scale bar = 100µm. (**D, E**) Bar graphs showing quantification of western blot analysis of the effect of mock or Lenti-Glut1 transduction on

TGF-β1-mediated changes in E-cadherin (**D**) and vimentin (**E**) levels. Data represent means±s.e.m. with three independent experiments in triplicates. *p<0.05.

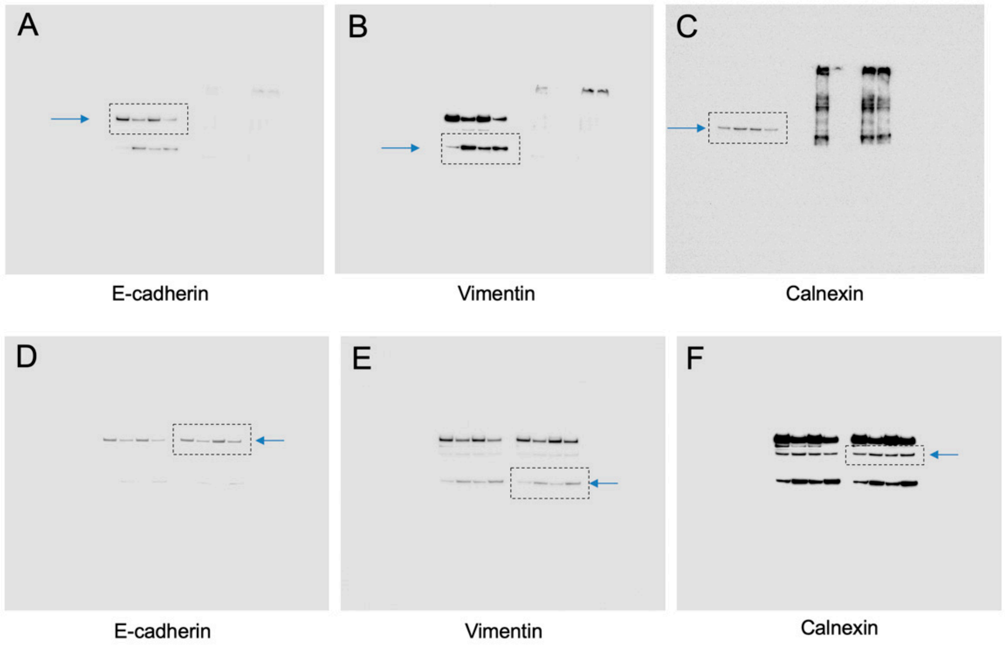

Figure S4. Original blots. Original blots for E-cadherin, vimentin and calnexin results included in Fig. 4B (A-C) and 4D (D-E).
